# The interaction of oxytocin and nicotine addiction on psychosocial stress: an fMRI study

**DOI:** 10.1038/s41398-024-03016-5

**Published:** 2024-08-30

**Authors:** Jiecheng Ren, Yuting Zhang, Hongwen Song, Huixing Gou, Qian Zhao, Wei Hong, Yi Piao, Yucan Chen, Yijun Chen, Shilin Wen, Zhangxin Du, Chuanfu Li, Bensheng Qiu, Yina Ma, Xiaochu Zhang, Zhengde Wei

**Affiliations:** 1 Department of Radiology, the First Affiliated Hospital of USTC, School of Life Science, Division of Life Science and Medicine, University of Science & Technology of China, Hefei, 230027 China; 2 Department of Psychology, School of Humanities & Social Science, University of Science & Technology of China, Hefei, Anhui 230026 China; 3 Application Technology Center of Physical Therapy to Brain Disorders, Institute of Advanced Technology, University of Science & Technology of China, Hefei, 230031 China; 4 Laboratory of Digital Medical Imaging, Medical Imaging Center, The First Affiliated Hospital of Anhui University of Traditional Chinese Medicine, Hefei, 230012 China; 5 Centers for Biomedical Engineering, School of Information Science and Technology, University of Science and Technology of China, Hefei, 230027 China; 6 State Key Laboratory of Cognitive Neuroscience and Learning IDG/McGovern Institute for Brain Research, Beijing Normal University, Beijing, 100091 China; 7 Business School, Guizhou Education University, Guiyang, 550018 China

**Keywords:** Addiction, Human behaviour

## Abstract

The anxiolytic effect of oxytocin (OXT) on psychosocial stress has been well documented, but the effectiveness under the interference of other factors still requires in-depth research. Previous studies have shown that nicotine addiction interacts with OXT on psychosocial stress on the behavioral level. However, the underlying neural mechanism of interaction between OXT and nicotine addiction on psychosocial stress has not been examined, and we conducted two experiments to reveal it. Firstly, after intranasal administration of randomized OXT or placebo (saline), a group of healthy participants (n = 27) and a group of smokers (n = 26) completed the Montreal Imaging Stress Task (MIST) in an MRI scanner. Secondly, a group of smokers (n = 22) was recruited to complete a transcranial direct current stimulation (tDCS) experiment, in which anodal tDCS was applied on subjects’ anterior right superior temporal gyrus (rSTG). In both experiment, subjective stress ratings, salivary cortisol samples and the amount of daily cigarette consumption were obtained from each participant. Analysis of variance were applied on both behavioral and neural data to examine the effects of OXT and nicotine addiction, and correlation analysis were used to examine relationships between neural and behavioral data. In first fMRI experiment, analysis of variance (ANOVA) revealed an interaction of OXT and nicotine addiction on subjective stress. In smokers, OXT failed to suppress the elevation of subjective stress and craving ratings after psychosocial stress. A voxel-wise ANOVA of fMRI data identified an interaction between OXT and nicotine addiction in anterior rSTG, and its functional connectivity with right middle frontal gyrus. Correlations between this functional connectivity and subjective psychosocial stress were also found abnormal in smokers. In second tDCS experiment, we found that under tDCS, OXT successfully suppressed the elevation of subjective stress and craving ratings after stress. In summary, we found that nicotine addiction blocked OXT’s anxiolytic on psychosocial stress, which was related to abnormalities in anterior rSTG. By applying anodal tDCS on anterior rSTG, OXT’s anxiolytic effect was restored in smokers. These findings will support further development on oxytocin’s intervention of psychosocial stress in nicotine addiction, and provides essential information for indicating OXT’s effectiveness.

## Introduction

The anxiolytic effect of oxytocin (OXT) on psychosocial stress has been well documented. Acute intranasal administration of oxytocin reduces both subjective and neuroendocrine responses to psychosocial stress [1, 2]. However, there is still a need for further in-depth research to fully understand the effectiveness of OXT on psychosocial stress [3]. Additionally, various factors, such as substance use, can interact with OXT’s anxiolytic effect [4, 5]. Investigating the neural basis of these factors can shed light on the neural mechanisms of OXT and help with OXT’s modulation for psychosocial stress.

Nicotine addiction is one of the factors that interacts with OXT on psychosocial stress. In nicotine addicts, craving is often provoked by psychosocial stress, and responses to psychosocial stress are also affected by nicotine addiction. Smokers typically have a blunted cortisol response compared to non-smokers in response to psychosocial stress [6]. Another study has shown that smokers have a disordered subjective anxiety response to acute stress [7]. Since OXT and nicotine addiction both impact the psychosocial stress system, nicotine addiction has the potential to interact with OXT. Recent studies have shown that the administration of OXT did not attenuate stress reactivity or subjective responses to smoking compared to a placebo. These findings indicate an alteration of OXT’s anxiolytic effect by nicotine addiction in smokers [8, 9]. Investigating the effects and interaction of OXT and nicotine addiction is essential in determining the effectiveness of OXT modulation for psychosocial stress, particularly for smokers. However, there is currently a lack of investigations on both neural and behavioral levels.

Previous research revealed that intranasal oxytocin alters activity in a large area of the brain’s social and stress system in psychosocial stress-inducing tasks, including the limbic area, medial prefrontal cortex and temporal cortex [10–12]. It has been suggested that OXT regulates the sensitivity of the HPA axis under psychosocial stress conditions through its actions on the limbic area, which possess high levels of OXT receptors. Brain regions such as the prefrontal cortex and superior temporal gyrus (STG) have also been found to be altered by oxytocin. These regions are essential for processing social information and are likely associated with subjective feelings of psychosocial stress [13–16].

In the realm of nicotine addiction, research has shown that a significant portion of the brain is affected, particularly in areas such as the prefrontal cortex and superior temporal gyrus, with the extent of damage being associated with the number of cigarettes consumed [17]. Additionally, resting-state fMRI examinations have revealed heightened fractional amplitude of low-frequency fluctuation (fALFF) in regions such as the left limbic lobe, right superior temporal gyrus, and right prefrontal cortex [18]. It has also been observed that nicotine deprivation can alter neural activity in various brain areas during psychosocial tasks, such as the prefrontal cortex and precuneus [19]. These findings collectively suggest that nicotine addiction has a widespread impact on the brain.

Given these findings, we assumed that responses to psychosocial stress in nicotine addicts should be abnormal, and functions of the overlapping brain areas of OXT’s anxiolytic effect and nicotine addiction are essential for the effects and interaction of OXT and nicotine addiction. These overlapping areas include the prefrontal cortices, limbic lobes, and superior temporal gyrus. In present study, we first conducted a within-subject double-blinded fMRI experiment, in which we recruited a group of healthy individuals and a group of smokers. Through the collection of subjective and physiological responses to psychosocial stress, indexes of nicotine addiction, and fMRI data, we were able to examine the effects and interaction between OXT and nicotine addiction on both behavioral and neural responses to psychosocial stress. We further evaluated the relationships between behavioral and neural responses related to this interaction. Additionally, we conducted a second transcranial direct current stimulation (tDCS) experiment to provide more causal evidence of our results.

## Methods and materials

### Participants

Needed sample size was calculated using G*power 3.1 software. We assumed a medium effect size (Cohen’s f = 0.25). A total sample size of 36 subjects was needed to detect a reliable effect with an alpha probability of 0.05 in a repeated-measures ANOVA.

A total of 58 male adults (age 18-50) participated in our first fMRI study, including 28 healthy controls (HCs) and 30 smokers (SMOs). One healthy participant and 4 smokers were excluded due to excessive head movement (more than 10% repetition times (TRs) with large motion (≥0.3 mm) censored). A total of 22 male smokers (age 18-50) participated in our second tDCS experiment. This study was approved by the Human Research Ethics Committee of the University of Science and Technology of China, and written informed consent was obtained from all participants before the experiments were conducted.

### Inclusion and exclusion criteria

Healthy controls were excluded with any current or past psychiatric disorders, including a specific phobia or past alcohol or nicotine abuse. Smokers were required to meet the following criteria: (1) smoke an average of 10 cigarettes/day for at least 2 years and (2) score ≥ 2 in the Fagerstrom Test of Nicotine Dependence (FTND; [20]), verified by expired breath CO ( ≥ 5 ppm) before the experiments. Smokers with other mental illnesses, substance abuse, and cardiovascular disease were excluded. Participants with magnetic resonance contraindications were also excluded.

### Procedure

In the first fMRI experiment, each participant first completed an online questionnaire including the FTND, state-trait anxiety inventory (STAI), and additional demographic and eligibility questions [21]. Smokers were instructed to smoke one cigarette two hours before starting each experiment.

**Fig. 1:**
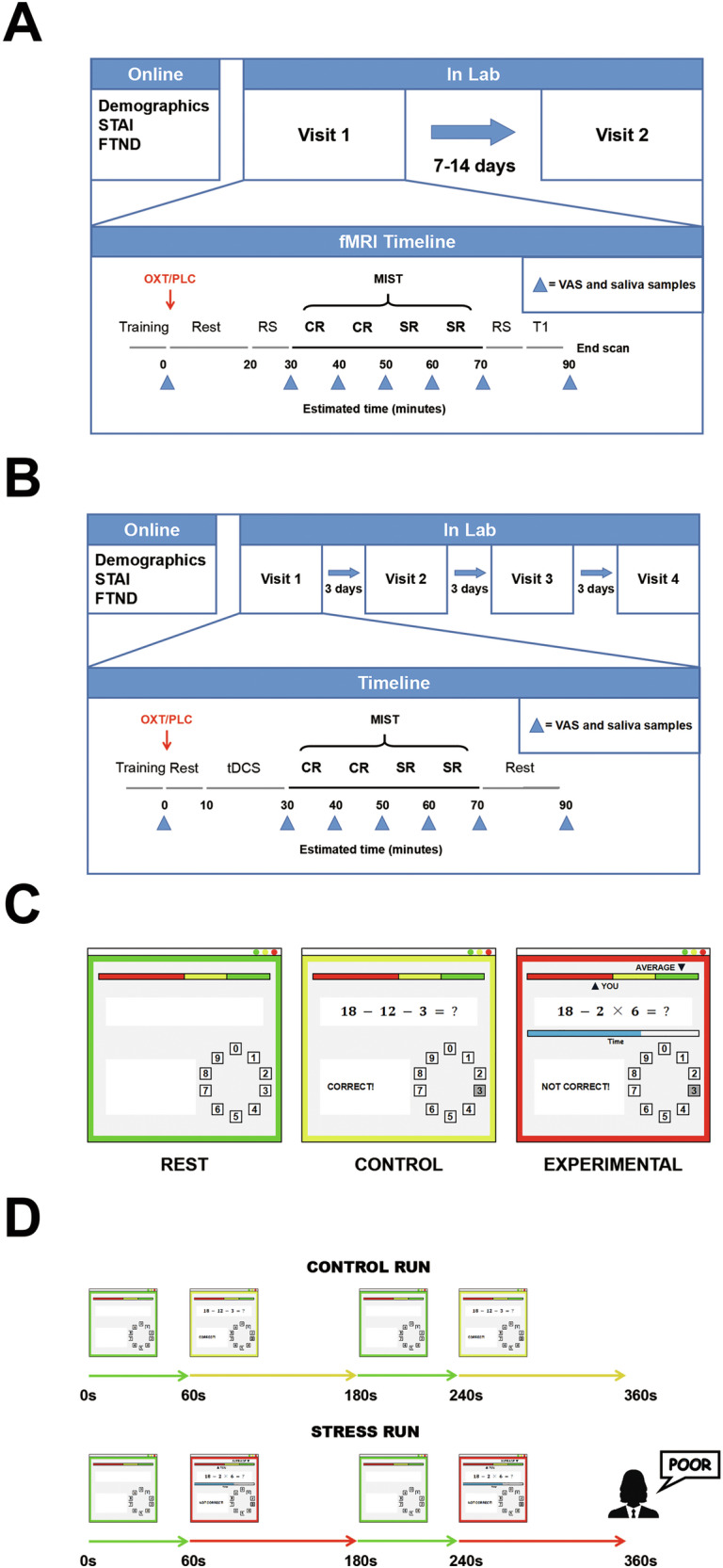
Design of the first fMRI experiment. Schematic diagram of the first fMRI experiment (**A**) and second tDCS experiment (**B**). The estimated time points of VAS, salivary sample collection and each MIST run are shown. The user interface of the Rest, Control and Experimental conditions of the MIST. **C** Each run consisted of two sets of the 60 s rest condition followed by 120 s math problem-solving condition. **D** STAI state-trait anxiety inventory, FTND Fagerstrom Test of Nicotine Dependence, MIST Montreal Imaging Stress Task, VAS visual analog scale, RS resting-state, CR control run, SR stress run, OXT oxytocin, PLC placebo, tDCS transcranial direct current stimulation.

Figure 1A depicts a schematic representation of the experimental procedure. All participants participated in two fMRI sessions in this double-blind randomized placebo-controlled within-subjects pharmaco-fMRI study. During each session, participants self-administered a single dose of 24 international units (IU) of random-ordered OXT (Defeng Pharmaceutical Co. Ltd, Sichuan, China) or a PLC nasal spray (Saline; Defeng Pharmaceutical Co. Ltd, Sichuan, China) consisting of three puffs per nostril prior to entering the scanner. The timing of treatment administration was based on previous pharmaco-kinetic studies [22], and occurred 45-55 minutes before stress induction.

The subsequent MRI acquisition included four Montreal Imaging Stress Task (MIST) runs lasting 6 min 30 s each, with 6-minutes-long resting-state scans performed both before and after the MIST runs [23]. After the first resting-state and four MIST runs, the scanner bed was moved out to collect participants’ subjective stress ratings and salivary samples.

To provide more evidence of results of the first fMRI experiment, we recruited a group of smokers and performed a second tDCS experiment. All participants completed four sessions in a double-blind randomized placebo-controlled within-subjects pharmaco-tDCS study. During each session, participants self-administered a single dose of 24 international units (IU) of random-ordered OXT (Defeng Pharmaceutical Co. Ltd, Sichuan, China) or a PLC nasal spray (Saline; Defeng Pharmaceutical Co. Ltd, Sichuan, China) consisting of three puffs per nostril before MIST task. After self-administration of nasal spray and 10 minutes of rest, a 20-minutes-long anodal tDCS or sham stimulation was random-ordered given to the participants. After the stimulation, participants completed four MIST runs, same as the first experiment. Figure 1B provides a schematic overview of the second experimental protocol. See more details in Supporting Information.

### Stress task design

Participants completed the Montreal Imaging Stress Task (MIST) paradigm while undergoing fMRI scanning [23]. The MIST is a computer program that displays a mental arithmetic task, a rotary dial for the submission of responses, and a text field that provides feedback on the submitted response. The task was block-designed and contained three conditions: Rest, Control, and Stress (Fig. 1C). Based on previous pharmaco-kinetic studies’ findings of intranasal oxytocin that changes it induced were sustained after treatment for over an hour (25-78 min), with a peak response at around 45 min [22], and other research which pointed out that oxytocin may facilitate active stress coping behaviors [44], participants were induced to psychosocial stress continuously for a period of 20 minutes, starting 45–55 minutes posttreatment. The paradigm comprised two control and two stress runs, consistent with previous studies [24, 25]. In addition, participants were instructed to complete one control run as a training run before each experiment. The composition of each run is shown in Fig. 1D.

During rest runs, participants focused on a screen displaying no task. In the control runs, participants engaged in arithmetic problem-solving by rotating the dial, with the understanding that their performance was not being recorded. In contrast, during the stress runs, participants were required to complete the problems within a limited timeframe. In the meantime, a performance that a participant could realistically achieve was described as the ‘average performance’ of the group, and a real-time performance comparison of the participants’ performance with ‘average performance’ was provided.

The initial difficulty level was determined based on participants’ accuracy during the training run. The MIST offers six levels of difficulty. For the training task, the difficulty was initially set at ‘level 4’ that involved arithmetic problems with up to four integers, including up to two two-digit numbers (example: 5*12-55). If participants achieved below 80% accuracy during the training task, the problem difficulty in the scanner was reduced to ‘level 3’. In the experimental condition, the difficulty was set at ‘level 4’, and the allotted time per problem was adjusted dynamically based on participants’ performance. The goal was to ensure that their accuracy remained low, but the task was still manageable enough for them to continue. During each experimental task, if participants had an accuracy above 50%, the time allowed per problem was decreased to increase task difficulty. Conversely, when accuracy fell below 50%, participants were given more time to solve the problems [23].

In the experimental condition, feedback for participants’ answers was presented (‘incorrect’, ‘correct’ or ‘out of time!’). They were instructed that the bar shown at the top of the interface represented their own performance and the performance of others with superior ‘average performance’. The MIST induced ‘achievement stress’ through this comparison. At the end of each run, researchers provided negative feedback, informing participants that their performance fell below average and encouraging them to pay closer attention and give their best effort. Participants were also reminded that there was a required minimum performance and his or her performance must be close or equal to average. Researchers also reminded participants that the investigator and colleagues were monitoring their performance even when outside the scanner room. After completing the experiment, participants were debriefed about the nature of the task and informed that they had actually performed well.

### Salivary cortisol

Salivary cortisol was collected as indicated in Fig. 1A, B. Samples were stored at -20°C until they were assayed for cortisol using an enzyme-linked immunosorbent assay (ELISA; assayed at Sangon Biotech Co., Ltd, Wuhan, China). See more details in Supporting Information.

### Subjective report ratings

Subjective stress and craving ratings were collected as indicated in Fig. 1A, B. All participants completed an adapted version of the visual analog scale (VAS). They were presented with a horizontal line representing a ten-point mood scale, in which 0 represented calm and 10 represented extremely stressed. Participants were instructed to provide a score between 0 and 10 that best described their current stress state. See more details in Supporting Information.

### Transcranial direct current stimulation

During the tDCS session, participants underwent 20 minutes of stimulation with a current intensity of 1.5 mA. The current was delivered by means of rubber electrodes embedded in sponges soaked in saline solution, which were secured in place using two rubber straps. In accordance with the EEG 10-20 system, the anodal electrode (4.5×6 cm=27 cm^2^) was positioned over the right anterior superior temporal gyrus (STG) at electrode site FT8. Conversely, the cathodal electrode (4.5 × 6 cm=27 cm^2^) was positioned above the left eye.

For anodal stimulations targeting the right anterior STG, a total of 1200 seconds (20 minutes) of continuous stimulation were administered, with an additional 30 seconds for gradual fade-in and another 30 seconds for fade-out. In the case of sham stimulation, a 30-second fade-in and a 30-second fade-out period were applied without any actual stimulation.

### Behavioral analyses

In first fMRI experiment, separate 2 (treatment: PLC and OXT) $\times$ 2 (group: HC and SMO) $\times$ 2 (time: stress run 1 and stress run 2) repeated-measures ANOVAs were used to identify the main and interaction effects of treatment, time and group on the changes in the subjective stress ratings and salivary cortisol levels. Regarding subjective craving ratings, paired t-test was used to measure the changes between OXT and PLC administration separately in stress run 1 and 2. In second tDCS experiment, separate 2 (treatment: PLC and OXT) $\times$ 2 (stimulation: tDCS and sham) $\times$ 2 (time: stress run 1 and stress run 2) repeated-measures ANOVAs were used to identify the main and interaction effects of treatment, time and stimulation on the changes in the subjective ratings and salivary cortisol levels. Further *post hoc* t-tests were conducted for within-subject and between-group difference detection. Analyses were conducted using SPSS (version 24.0; IBM SPSS Statistics, Armonk, NY, USA).

### fMRI data analyses

fMRI images were preprocessed and analyzed using the standard procedure in Analysis of Functional NeuroImages (AFNI; https://afni.nimh.nih.gov/; [26, 27]). Evoked blood oxygen level-dependent (BOLD) activity was estimated for each run and participant by constructing a general linear model (GLM) that convolved the regressors with the canonical hemodynamic response function (HRF).

A voxelwise 2 (Treatment: PLC and OXT) $\times$ 2 (Group: HC and SMO) $\times$ 2 (Time: stress run 1 and stress run 2) repeated-measures ANOVA (stress > control) was conducted on the whole brain [28]. The significance threshold was set to p$\le$ 0.001, and multiple comparisons were corrected using the program ‘3dFDR’ and ‘3dClustSim’ in AFNI. FDR-corrected threshold of p$\le$ 0.05 and a minimum cluster size of 60 voxels was set. Clusters with interactions were identified. After whole-brain voxelwise repeated-measures ANOVA was done, masks of regions of interest (ROIs) were created using Automated Anatomic Labeling (AAL) atlas. Coordinates of the center of mass of the four brain regions which had Treat $\times$ Group interaction were identified, and the corresponding four regions in AAL were extracted to create the ROI masks [29].

To inspect context-distinct variations in functional connectivity between ROIs and the rest of the brain, we conducted psychophysiological interaction (PPI) assessment at whole-brain level using anterior right STG as our seed region. Functional connectivities were Fisher-z transformed, and changes between two stress runs were pooled into a voxelwise 2 (Treatment: PLC and OXT) $\times \,$2 (Group: HC and SMO) $\times$ 2 (Time: stress run 1 and stress run 2) repeated-measures ANOVA for investigations for OXT and nicotine addiction’s effect on the functional connectivities (FDR-corrected threshold of p$\le$ 0.05 and a minimum cluster size of 60 voxels was set using program ‘3dFDR’ and ‘3dClustSim’). After that, *post hoc* t-tests were conducted for within-subject and between-group difference detection. See more details in Supporting Information.

### Associations between neural and behavioral or physiological data

Pearson correlations were calculated to investigate relationships between neural and behavioral data. Between-group correlation differences were tested using Fisher’s z-tests. The significance threshold was set at *p* < 0.05, Bonferroni corrected. See more details in Supporting Information.

## Results

### Demographics and smoking characteristics

A total of 27 healthy control participants (HCs) and 26 smokers (SMOs) completed two sessions of fMRI experiments. There were no significant differences between HCs and SMOs in age, years of education, or anxiety levels, as assessed by the State-Trait Anxiety Inventory (STAI). A total of 22 smokers completed four sessions of tDCS experiments. None of the participants reported awareness of the existence of a placebo. Indexes of nicotine addiction and demographic characteristics are shown in Table 1.

**Table 1:** Demographics and smoking characteristics.

	Experiment I			Experiment II
Characteristics	HC^a^ (n = 27) Mean ± SEM	SMO^b^ (n = 26) Mean ± SEM	p	SMO (n = 22) Mean ± SEM
**Age**	23.74 ± 0.57	25.42 ± 0.85	0.103	26.68 ± 1.41
**Education**	16.38 ± 0.38	15.65 ± 0.29	0.135	15.50 ± 0.53
**STAI-S**^**c**^	37.67 ± 1.84	39.92 ± 1.81	0.387	38.95 ± 1.84
**STAI-T**^**d**^	42.00 ± 1.77	45.96 ± 1.86	0.128	42.23 ± 1.85
**FTND**^**e**^	–	4.56 ± 0.37	–	5.05 ± 0.32
**CPD**^**f**^	–	21.41 ± 2.12	–	20.55 ± 0.87
**Smoking years**	–	7.96 ± 0.89	–	9.00 ± 1.16

^a^HC: Healthy control

^b^SMO: smokers

^c^STAI-S: State-Trait Anxiety Inventory-State

^d^STAI-T: State-Trait Anxiety Inventory-Trait

^e^FTND: Fagerstrom Test of Nicotine Dependence

^f^CPD: cigarettes per day

### Experiment I: Effects of oxytocin and nicotine addiction on subjective ratings and salivary cortisol

After each MIST run, we collected participants’ subjective stress ratings and samples of salivary cortisol. Changes in subjective stress ratings and salivary cortisol were included in 2 (Treatment: PLC and OXT) $\times$ 2 (Group: HC and SMO) $\times$ 2 (Time: stress run 1 and stress run 2) repeated-measures ANOVAs (N = 53).

**Fig. 2:**
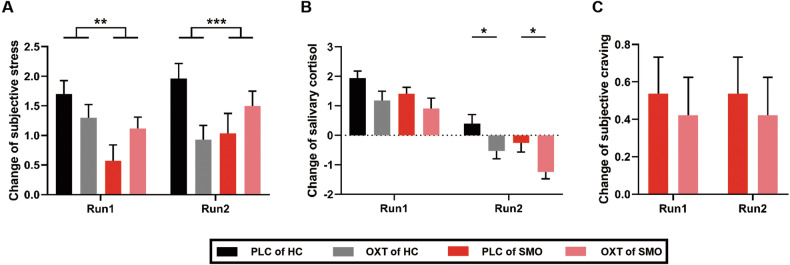
Effects of oxytocin and nicotine addiction on subjective ratings and salivary cortisol levels. Changes in subjective stress ratings (**A**), salivary cortisol levels (**B**) and subjective craving ratings (**C**) in stress run 1 and stress run 2. An interaction between OXT and nicotine addiction occurred in subjective stress but not in salivary cortisol. HC: healthy participants group (n = 27); SMO: smokers group (n = 26); PLC placebo, OXT oxytocin; *p < 0.05; **p < 0.01; ***p < 0.001.

There was a significant main effect of Time (F_(1, 51)_ = 6.340, *p* = 0.015), along with a significant Treatment $\times$ Group $\times$ Time interaction (F_(1, 51)_ = 4.512, *p* = 0.039; Fig. 2A) in changes of subjective stress. In stress run 1, in smokers, *post hoc* examinations revealed a larger elevation of subjective stress after OXT than after PLC (t_25_ = 3.275, *p* = 0.002). In healthy participants, there was a smaller elevation of subjective stress after OXT than after PLC administration (t_26_ = -2.576, *p* = 0.016). In stress run 2, smokers showed a larger elevation of subjective stress after OXT than PLC administration (t_25_ = 2.478, *p* = 0.020). In healthy participants, there was a smaller elevation of subjective stress after OXT than PLC administration (t_26_ = -3.502, *p* = 0.002). Examinations of between-group difference revealed a smaller elevation of subjective stress after PLC administration in smokers than in healthy participants in stress run 2 (t_51_ = 2.449, *p* = 0.018).

After collecting subjective stress ratings, we collected participants’ salivary cortisol samples. We observed significant main effects of treatment (F_(1, 51)_ = 14.598, *p* < 0.001), time (F_(1, 51)_ = 95.540, *p* < 0.001) and group (F_(1, 51)_ = 5.428, *p* = 0.024), but no significant interaction. *Post hoc* examinations showed no significant within-subject or between-group differences in salivary cortisol changes in stress run 1. In stress run 2, there were significant drops in salivary cortisol after OXT administration, both in healthy participants (t_26_ = -2.574, *p* = 0.016) and smokers (t_25_ = -2.686, *p* = 0.013; Fig. 2B).

Regarding subjective craving ratings, we found no difference between OXT and PLC administration, both in stress run 1 or 2 (Fig. 2C).

### Experiment I: Effects of oxytocin and nicotine addiction on psychosocial stress in the whole brain

**Fig. 3:**
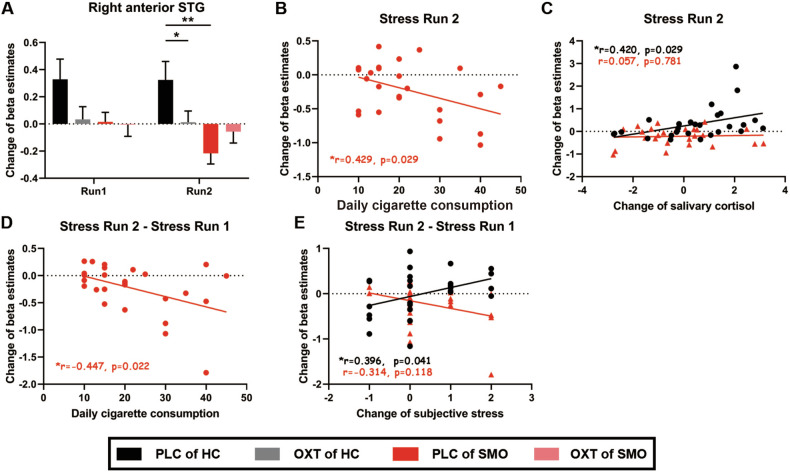
Effects of oxytocin and nicotine addiction on psychosocial stress in anterior superior temporal gyrus. Changes in anterior right superior temporal gyrus (rSTG) activation in stress runs 1 and 2 (**A**). The changes in beta estimates of the anterior rSTG in stress run 2 were inversely correlated with an index of nicotine addiction after PLC administration (**B**). In the HC group, changes in beta estimates of the anterior rSTG were positively associated with changes in salivary cortisol after the administration of PLC in stress run 2 (**C**). In the SMO group, between-run changes in beta estimates of the anterior rSTG were inversely correlated with an index of nicotine addiction after PLC administration (**D**). Between-runs changes in the beta value of the anterior rSTG were positively associated with changes in subjective stress ratings after administration of PLC, which was significantly differently directed in the SMO group (**E**). HC: healthy participants group (n = 27); SMO: smoker group (n = 26); PLC: placebo; OXT: oxytocin; *p < 0.05; **p < 0.01.

A voxelwise 2 (Treatment: PLC and OXT) $\times$ 2 (Group: HC and SMO) $\times$ 2 (Time: stress run 1 and stress run 2) repeated-measures ANOVA (stress > control) was conducted on the whole brain. There were significant Treatment $\times$ Group interactions in four brain regions: the anterior right superior temporal gyrus (rSTG), medial frontal gyrus, right lentiform nucleus / inferior frontal gyrus, and right parahippocampal gyrus (FDR corrected *p* < 0.05 with a minimum cluster size of 60 voxels; Table 2; Fig. S1). Neural activities of these four brain regions were extracted by using AAL-defined masks. Examinations of difference showed that in stress run 2, changes in anterior rSTG activity in healthy participants were lower after OXT than after PLC administration (t_26_ = -2.057, *p* = 0.050). Examinations of group difference showed that changes in anterior rSTG activity were lower in smokers than in healthy participants after PLC administration (t_51_ = -3.435, *p* = 0.001; Fig. 3A), and in a different direction of activation. The results of the ROI analysis of the other three brain regions (medial frontal gyrus, right lentiform nucleus/right inferior frontal gyrus and right parahippocampal gyrus) are shown in Fig. S2.

**Table 2:** Brain areas revealed significant Treatment $\times \,$Group interactions.

Region^a^	Voxels	CMass^b^ x^c^	CMass y	CMass z	Peak z
***Treatment × Group***					
**R anterior superior temporal g**^**d**^	274	−40	−14	−22	5.199
**Medial frontal g**	263	−1	−42	+25	4.866
**R lentifrom nucleus/R Inferior frontal g**	167	−29	−17	−1	4.387
**R parahippocampal g**	123	−28	+19	-17	4.775

^a^Clusterwise corrected ≥ 60 voxels, corrected *p* < 0.05.

^b^CMass: center of mass.

^c^Talaraich/Tourneaux coordinates.

^d^g: gyrus. Regions listed survived multiple-comparisons correction.

Next, we conducted correlation analysis to investigate relationships between the neural activities and the behavioral data. In stress run 2, changes in anterior rSTG activity were inversely associated with daily cigarette consumption after administration of PLC in smokers (r = -0.429, *p* = 0.029; Fig. 3B). Changes in anterior rSTG activity in stress run 2 were positively associated with changes in salivary cortisol after administration of PLC in healthy participants (r = 0.420, *p* = 0.029; Fig. 3C). Associations between neural activity and behavioral data including indexes of nicotine addiction and psychosocial stress were not observed simultaneously in the medial frontal gyrus, right lentiform nucleus/right inferior frontal gyrus or right parahippocampal gyrus.

We also investigated the changes in anterior rSTG activity between two stress runs and their relationships with behavioral data. In stress run 2, changes in anterior rSTG activity were inversely associated with daily cigarette consumption after administration of PLC in smokers (r = -0.447, *p* = 0.022; Fig. 3D). Changes in anterior rSTG activity were positively associated with changes in subjective stress ratings when treated with PLC in healthy participants(r = 0.396, *p* = 0.041). This correlation was significantly differently directed in smokers (Fisher’s z = 2.55, *p* = 0.022, two-tailed; Fig. 3E).

### Experiment I: Effects of oxytocin and nicotine addiction on anterior rSTG’s functional connectivity with stress-related brain regions

**Fig. 4:**
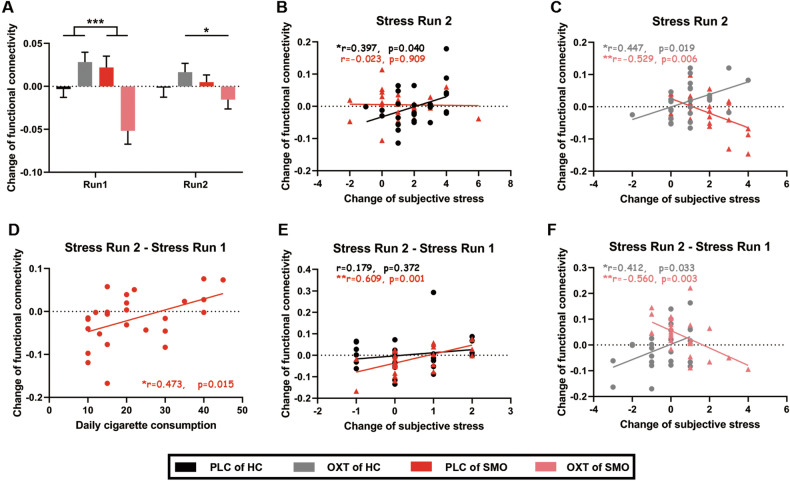
Effects of oxytocin and addiction on the functional connectivity between the anterior superior temporal gyrus and right middle frontal gyrus. Changes in functional connectivity between the anterior rSTG and right middle frontal gyrus (rMFG) in stress runs 1 and 2 (**A**). In o 2, changes in the coupling of the anterior rSTG and rMFG were positively associated with changes in subjective stress in healthy participants after the administration of PLC (**B**). After OXT administration, changes in functional connectivity between the anterior rSTG and rMFG showed differently directed associations with changes in subjective stress ratings in HCs and smokers (**C**). Between-run changes in anterior rSTG–rMFG coupling were positively associated with daily cigarette consumption in smokers after PLC administration (**D**). After the administration of PLC, between-run changes in functional connectivity between the anterior rSTG and rMFG showed the same directed but nearly different associations with changes in subjective stress ratings in the HC and SMO groups (**E**) but showed differently directed associations with changes in subjective stress ratings in HCs and smokers after the administration of OXT (**F**). HC: healthy participants group (n = 27); SMO: smokers group (n = 26); PLC: placebo; OXT: oxytocin; *p < 0.05; **p < 0.01.

We performed psychophysiological interaction (PPI) analysis using the anterior rSTG as the seed regions at the whole-brain level. A voxelwise 2 (Treatment: PLC and OXT) $\times$ 2 (Group: HC and SMO) $\times$ 2 (Time: stress run 1 and stress run 2) repeated-measures ANOVA revealed a significant main effect of Group in anterior rSTG’s functional connectivities with right thalamus (rTHA, peak coordinates in the talaraich space: [-17, 19, 2]). A significant Treatment $\times$ Group interaction was also detected in right precuneus (rPCu, peak coordinates in the talaraich space: [-11, 61, 26]) and right middle frontal gyrus (rMFG, peak coordinates in the talaraich space: [-23, -17, 38]; FDR corrected *p* < 0.05 with a minimum cluster size of 60 voxels). Subsequent difference examinations revealed that in stress run 1, OXT administration significantly strengthened anterior rSTG-rMFG connectivity in healthy participants (t_26_ = 2.464, *p* = 0.021) but weakened this connectivity in smokers (t_25_ = -4.124, *p* < 0.001), compared with PLC. This effect was not significant in stress run 2. Examinations of between group difference revealed that in both stress runs, compared with healthy participants, changes in coupling between the anterior rSTG and rMFG were lower in smokers after OXT administration (stress run 1: t_51_ = -4.165, *p* < 0.001; stress run 2: t_51_ = -2.147, *p* = 0.037; Fig. 4A). Changes in coupling between the anterior rSTG and rPCu are shown in Fig. S3A.

Next, we conducted correlation analysis to examine the relationships between the coupling of the anterior rSTG and rMFG and behavioral data. In stress run 2, changes in the coupling of the anterior rSTG and rMFG were positively associated with changes in subjective stress in healthy participants, both after administration of OXT (r = 0.447, *p* = 0.019) or PLC (r = 0.397, *p* = 0.040). In smokers, changes in the coupling of the anterior rSTG and rMFG were inversely associated with subjective stress after the administration of OXT (r = -0.529, *p* = 0.006), with a significant difference from the healthy participants (Fisher’s z = 3.67, *p* < 0.001, two-tailed; Fig. 4B, C). Notably, in stress run 2, changes in the coupling of the anterior rSTG and rMFG in smokers were nearly significantly associated with daily cigarette consumption after PLC administration (r = 0.353, *p* = 0.077).

No association was observed between the coupling of the anterior rSTG and rPCu and indices of nicotine addiction (*p* > 0.1). A positive association was observed between changes in salivary cortisol and changes in the coupling of the anterior rSTG and rPCu after OXT administration when healthy participants and smokers were combined (r = 0.290, *p* = 0.035); a trend of difference with the administration of PLC (r = -0.040, *p* = 0.773) was observed (z = 1.69, *p* = 0.091, two-tailed; Figure S3B).

Further, we calculated changes in the coupling of the anterior rSTG and rMFG between two stress runs and examined their relationships with behavioral data. In smokers, we found that changes in coupling of anterior rSTG and rMFG after administration of PLC were positively correlated with daily cigarette consumption (r = 0.473, *p* = 0.015; Fig. 4D). After PLC administration, changes in the coupling of the anterior rSTG and rMFG between two stress runs were positively associated with changes in subjective stress ratings in the smoker group (r = 0.609, *p* = 0.001), and the direction was the same with HC group and the difference of correlations was nearly significant (Fisher’s z = 1.80, *p* = 0.072, two-tailed; Fig. 4E). In smokers, changes in the coupling of the anterior rSTG and rMFG between two stress runs were negatively associated with changes in subjective stress ratings after treatment with OXT (r = -0.560, *p* = 0.003). In healthy participants, this correlation was positively associated (r = 0.412, *p* = 0.033), and the between-group difference was significant (Fisher’s z = 3.67, *p* < 0.001; Fig. 4F).

### Experiment II: Effects of oxytocin and rSTG-tDCS on subjective stress ratings and salivary cortisol in smokers

Next, we conducted a tDCS experiment to investigate the causal relationship between the anterior rSTG and OXT’s impact on psychosocial stress in smokers. Employing anodal tDCS over the anterior rSTG, our aim was to restore the activity of this region in response to psychosocial stress, thereby reinstating OXT’s anxiolytic effect on subjective stress.

**Fig. 5:**
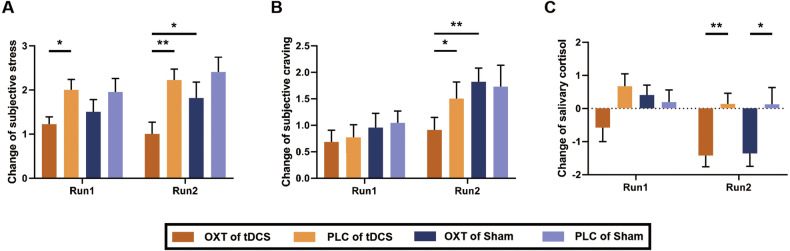
Effects of oxytocin and rSTG-tDCS on subjective ratings and salivary cortisol levels in smokers. Changes in subjective stress ratings (**A**), subjective craving ratings (**B**) and salivary cortisol level (**C**) in stress run 1 and stress run 2 in the tDCS experiment. tDCS: transcranial direct current stimulation; PLC: placebo; OXT: oxytocin; *p < 0.05; **p < 0.01.

Changes in subjective stress ratings, subjective craving ratings and salivary cortisol were included in 2 (Treatment: PLC and OXT) $\times$ 2 (Stimulation: tDCS and sham) $\times$ 2 (Time: stress run 1 and stress run 2) repeated-measures ANOVAs (N = 23). Regarding changes in subjective stress, we observed a significant main effect of Time (F_(1, 21)_ = 8.225, *p* = 0.009), accompanied by a significant Stimulation × Time interaction (F_(1, 21)_ = 5.319, *p* = 0.031). Subsequent *post hoc* analyses revealed a diminished increase in subjective stress following OXT administration compared to PLC administration, both during stress run 1 (t_21_ = -2.631, *p* = 0.016) and stress run 2 (t_21_ = -3.594, *p* = 0.002; Fig. 5A). Notably, during stress run 2, the elevation of subjective stress was significantly lower after OXT administration combined with tDCS compared to sham stimulation (t_21_ = -2.673, *p* = 0.014).

The analysis of changes in subjective craving ratings unveiled significant main effects of Stimulation (F_(1, 21)_ = 4.375, *p* = 0.050) and Time (F_(1, 21)_ = 17.447, *p* < 0.001). Specifically, in stress run 2, *post hoc* assessments demonstrated a reduced escalation of subjective craving following OXT administration compared to PLC administration after tDCS (t_21_ = -2.200, *p* = 0.039; Fig. 5B). Moreover, following OXT administration, the increase in subjective craving was notably smaller after tDCS compared to sham stimulation (t_21_ = -4.004, *p* = 0.001).

In the evaluation of salivary cortisol changes, significant main effects were observed for Treatment (F_(1, 21)_ = 10.472, *p* = 0.030) and Time (F_(1, 21)_ = 16.934, *p* < 0.001), along with a significant Treatment $\times$ Stimulation $\times$ Time interaction (F_(1, 21)_ = 4.816, *p* = 0.040). Specifically, during stress run 2, a substantial reduction in salivary cortisol levels was evident following OXT administration when compared to PLC administration, both after tDCS (t_21_ = -3.720, *p* = 0.001; Fig. 5C) and sham stimulation (t_21_ = -2.815, *p* = 0.010).

## Discussion

The current study utilized fMRI to investigate both the behavioral and neural effects of OXT and nicotine addiction on psychosocial stress. Our findings revealed an interaction between OXT and nicotine addiction on subjective psychosocial stress, and identified four brain regions associated with this interaction. Specifically, the anterior rSTG and its functional connectivity with the rMFG were found to be associated with behavioral psychosocial stress.

Previous research has suggested that OXT can suppress salivary cortisol and subjective responses to psychosocial stress in healthy individuals [2]. However, in smokers, the anxiolytic effect of OXT was found to be weakened in several studies [8, 9]. Consistent with these findings, our study demonstrated a similar weakened anxiolytic effect of OXT in smokers, with no reduction in subjective stress ratings. This may be due to dysregulated stress responses in nicotine addiction, as smokers often demonstrate a blunted cortisol response compared to non-smokers [6]. Further, acute stress has been reported to have no effect on subjective anxiety in smokers, and subjective anxiety has been found to be dysfunctional after acute stress [7, 30]. It is possible that a dysregulated stress system could lead to abnormal OXT function in smokers, as the brain stress system overlaps with the brain system that oxytocin regulates.

The role of the rSTG in both brain stress and social systems has been widely studied. For instance, one meta-analysis has shown that activation in the rSTG may distinguish psychosocial stress from physiological stress [31]. In the current study, we identified the anterior rSTG as a site of interaction between OXT and nicotine addiction on psychosocial stress, as well as a brain region related to psychosocial stress and nicotine addiction. The right STG plays a crucial role in social information processing, with the posterior rSTG processing detailed sensory information and the anterior rSTG processing social conceptual information [32, 33]. Moreover, the right STG is an important part of the oxytocin-mediated prosocial neural circuit. A study has shown that OXT enhances activation in the rSTG and improves participants’ ability to process social-related information [34]. Additionally, neural activity in the rSTG was found to be altered under psychosocial stress after OXT administration [10]. Therefore, alterations in this brain region have the potential to affect OXT’s regulation of psychosocial stress.

The anterior STG plays a crucial role in processing social information, and thus any alterations in the anterior rSTG may impact social information processing [35]. The Montreal Imaging Stress Task was designed to induce psychosocial stress in the context of a scanning environment. According to developer of this task, social evaluative threat information as feedback from the task itself and the investigator was built into the task [23]. In the current study, we found that the effect of OXT on the anterior rSTG under psychosocial stress was altered in smokers, likely due to the dysregulated stress responses caused by nicotine addiction, which can lead to abnormal OXT function. According to previous investigations, nicotine addiction damages a range of brain regions, including the anterior rSTG, while our results also showed that anterior rSTG activation changes were correlated with daily cigarette consumption [36]. Combining this evidence and our results, the alteration of the anterior rSTG by nicotine addiction in smokers may lead to an altered self-processing of information related to subjective feelings of stress, such as interactions with OXT on psychosocial stress. The altered anterior rSTG in smokers may lead to an altered self-processing of information related to subjective feelings of stress, such as interactions with OXT on psychosocial stress. In our second tDCS experiment, we initially replicated the findings from the fMRI experiment concerning smokers’ subjective feelings and salivary cortisol levels within our sham stimulation group. Subsequently, our focus shifted to the application of anodal tDCS on the anterior rSTG with the objective of reinstating its responsiveness to psychosocial stress. The outcomes of this experiment revealed that, under the influence of tDCS, OXT effectively mitigated the increase in both subjective stress and craving ratings, as well as salivary cortisol levels. These results offer empirical support from a bottom-up perspective, reinforcing the pivotal role of the anterior rSTG in shaping subjective perceptions of stress, particularly among individuals who smoke.

Our PPI analysis revealed interactions between Treatment and Group in the coupling of the anterior rSTG with the right precuneus and rMFG, two brain regions involved in social information perception [37, 38]. The middle frontal gyrus is a known site for attention and working memory [39] and can be equipped for identified salience [40]. The persistence of MIST stress runs may strengthen the salience of social stress information, thus increasing perceived stress. By regulating the coupling of the anterior rSTG and rMFG, OXT could regulate the salience of social stress and induce successful coping by suppressing subjective ratings [41]. rMFG’s function is potentially vulnerable in smokers as it is impaired in nicotine addiction [42]. In present study, the direction of the coupling of anterior rSTG and rMFG and subjective stress differs between smokers and healthy participants. This altered relationship in smokers may indicate the neural mechanism by which OXT and nicotine addiction interact with each other in the coupling of the anterior rSTG and rMFG, thus affect subjective stress.

We found that the relationship between neural activity or functional connectivity and behavioral indexes of psychosocial stress mainly appeared in stress run 2 and changes between the two stress runs. This may be due to oxytocin’s effect on coping behavior. Oxytocin has been found to switch passive stress-coping behavior, such as freezing, to active stress-coping mechanisms such as flight when facing an imminent stressor [43]. In one human study, oxytocin induced quick adaptations to frightening stimuli in aversive contexts [44]. This regulation of changing coping strategy is referred to as allostasis of stress [3]. Oxytocin is not simply anxiolytic but induces rapid and flexible adaptation to aversive situations. On the other hand, oxytocin has also been found to increase the resilience of stress. In an animal study, oxytocin receptor-deficient mice showed no increased defeat posturing during repeated social defeat stress [45]. This suggests that oxytocin may facilitate resilience of stress by induce coping behavior. The relationship between anterior rSTG neural activity or anterior rSTG-rmFG coupling and subjective stress in healthy participants occurring in stress run 2 or between two stress runs reveals part of the neural mechanisms of subjective stress coping. However, this correlation was reversed in smokers after OXT administration, suggesting that the interaction between nicotine addiction and OXT occurred in the process of subjective psychosocial stress coping, related to the anterior rSTG and its connectivity with the rMFG.

In the present study, although changes in anterior rSTG activity were mainly associated with changes in subjective stress ratings during the two stress runs, in stress run 2, changes in anterior rSTG activity were also associated with salivary cortisol in healthy participants after PLC administration. The temporal lobe is closely connected with self-directed social information processing [46], but previous studies have also pointed to the associations between physiological representations of stress and the temporal lobe [47]. In the present study, in a sample of healthy participants and smokers, after OXT administration, changes in the coupling of the anterior rSTG-rPCu were associated with changes in salivary cortisol. The precuneus is part of the brain default mode network (DMN), and a stress-induced cortisol increase was found to be associated with increased connectivity within the salience network but with decreased coupling of the DMN both within and outside the network [48]. OXT administration altered the connectivity strength of the rPCu and may have altered this association.

Functional neuroimaging studies have been criticized for providing correlational but not causal information. However, the present study provides a causal perspective on the neural mechanisms of OXT’s regulation of psychosocial stress. In first fMRI experiment, OXT failed to suppress subjects’ subjective feelings of psychosocial stress and the rSTG is differently damaged in smokers compared with healthy participants. But, by applying tDCS upon anterior rSTG on smokers, we restored OXT’s anxiolytic effect, which provides essential causal information about the relationship of anterior rSTG and subjective feelings related to psychosocial stress in smokers. OXT has been widely used as an modulation for psychosocial stress, but the effectiveness of OXT still requires further in-depth research [3]. In this sense, functional integrity of the anterior rSTG could be an indicator of the effectiveness of OXT.

Although this study sheds new light on the effects of OXT and nicotine addiction on psychosocial stress, it has some limitations. A single dose of OXT was administered in each experiment, and future studies are needed to investigate the neural mechanisms of the long-term effects of OXT. Additionally, early life stress and social support were not controlled for in this study, although recent research has shown that they can cause individual differences in OXT’s effects [49]. Finally, we only tested male participants in present study due to the huge disparity in the male-to-female smoker ratio in China, and since OXT has sex-dimorphic effects, the exploration of OXT’s effect on women is lacking.

In summary, our study provides novel behavioral and neural information on the effects and interaction of OXT and nicotine addiction on psychosocial stress. Our findings suggest that nicotine addiction blocks OXT’s anxiolytic effect on psychosocial stress, which is related to abnormalities in the anterior rSTG. These results have important implications for the development of interventions for psychosocial stress and for assessing the effectiveness of OXT, particularly in smokers.

### Supplementary information

The interaction of oxytocin and nicotine addiction on psychosocial stress: an fMRI study

## Data Availability

The data that support the findings of this study are available from the corresponding author, Z. D. W and X. C. Z, upon reasonable request.

## Code availability

The data and code supporting the findings of this study are available on request from the corresponding author.

## References

[1] de Oliveira DCG, Zuardi AW, Graeff FG, Queiroz RHC, Crippa JAS. Anxiolytic-like effect of oxytocin in the simulated public speaking test. J Psychopharmacol Oxf Engl. 2012;26:497–504. 10.1177/0269881111400642 21555332

[2] Heinrichs M, Baumgartner T, Kirschbaum C, Ehlert U. Social support and oxytocin interact to suppress cortisol and subjective responses to psychosocial stress. Biol Psychiatry. 2003;54:1389–98. 14675803 10.1016/S0006-3223(03)00465-7

[3] Takayanagi Y, Onaka T. Roles of oxytocin in stress responses, allostasis and resilience. Int J Mol Sci. 2021;23:150. 35008574 10.3390/ijms23010150 PMC8745417

[4] Chen FS, Kumsta R, von Dawans B, Monakhov M, Ebstein RP, Heinrichs M. Common oxytocin receptor gene (OXTR) polymorphism and social support interact to reduce stress in humans. Proc Natl Acad Sci USA. 2011;108:19937–42. 22123970 10.1073/pnas.1113079108 PMC3250137

[5] Sanna F, De Luca MA. The potential role of oxytocin in addiction: what is the target process? Curr Opin Pharmacol. 2021;58:8–20. 33845377 10.1016/j.coph.2021.03.002

[6] al’Absi M, Wittmers LE, Erickson J, Hatsukami D, Crouse B. Attenuated adrenocortical and blood pressure responses to psychological stress in ad libitum and abstinent smokers. Pharmacol Biochem Behav. 2003;74:401–10. 12479961 10.1016/S0091-3057(02)01011-0

[7] Woodcock EA, Stanley JA, Diwadkar VA, Khatib D, Greenwald MK. A neurobiological correlate of stress-induced nicotine-seeking behavior among cigarette smokers. Addict Biol. 2020;25:e12819. 31418989 10.1111/adb.12819 PMC7023991

[8] McClure EA, Baker NL, Gray KM, Hood CO, Tomko RL, Carpenter MJ, et al. The influence of gender and oxytocin on stress reactivity, cigarette craving, and smoking in a randomized, placebo-controlled laboratory relapse paradigm. Psychopharmacology (Berl). 2020;237:543–55. 31792646 10.1007/s00213-019-05392-z PMC7024045

[9] Van Hedger K, Bershad AK, Lee R, de Wit H. Effects of intranasal oxytocin on stress-induced cigarette craving in daily smokers. Nicotine Tob Res. 2020;22:89–95. 30085292 10.1093/ntr/nty159 PMC7297012

[10] Chen X, Hackett PD, DeMarco AC, Feng C, Stair S, Haroon E, et al. Effects of oxytocin and vasopressin on the neural response to unreciprocated cooperation within brain regions involved in stress and anxiety in men and women. Brain Imaging Behav. 2016;10:581–93. 26040978 10.1007/s11682-015-9411-7 PMC4670292

[11] Grimm S, Pestke K, Feeser M, Aust S, Weigand A, Wang J, et al. Early life stress modulates oxytocin effects on limbic system during acute psychosocial stress. Soc Cogn Affect Neurosci. 2014;9:1828–35. 24478326 10.1093/scan/nsu020 PMC4221227

[12] Rolls ET. The cingulate cortex and limbic systems for emotion, action, and memory. Brain Struct Funct. 2019;224:3001–18. 31451898 10.1007/s00429-019-01945-2 PMC6875144

[13] Mellem MS, Jasmin KM, Peng C, Martin A. Sentence processing in anterior superior temporal cortex shows a social-emotional bias. Neuropsychologia. 2016;89:217–24. 27329686 10.1016/j.neuropsychologia.2016.06.019 PMC5384858

[14] Wood JN. Social cognition and the prefrontal cortex. Behav Cogn Neurosci Rev. 2003;2:97–114. 13678518 10.1177/1534582303002002002

[15] Ma M, Chang X, Wu H. Animal models of stress and stress-related neurocircuits: a comprehensive review. Stress Brain. 2021;1:108–27. 10.26599/SAB.2021.9060001

[16] Liu X, Yu C, Yu H-H, Chen Z, Zhou D. The cognitive function effects of prefrontal tDCS for depression: a system review. Stress Brain. 2021;1:97–107. 10.26599/SAB.2020.9060006

[17] Fritz H-C, Wittfeld K, Schmidt CO, Domin M, Grabe HJ, Hegenscheid K, et al. Current smoking and reduced gray matter volume-a voxel-based morphometry study. Neuropsychopharmacol Off Publ Am Coll Neuropsychopharmacol. 2014;39:2594–2600. 10.1038/npp.2014.112 PMC4207339 24832823

[18] Chu S, Xiao D, Wang S, Peng P, Xie T, He Y, et al. Spontaneous brain activity in chronic smokers revealed by fractional amplitude of low frequency fluctuation analysis: a resting state functional magnetic resonance imaging study. Chin Med J (Engl). 2014;127:1504–9. 24762597 10.3760/cma.j.issn.0366-6999.20131608

[19] Ashare RL, Lerman C, Cao W, Falcone M, Bernardo L, Ruparel K, et al. Nicotine withdrawal alters neural responses to psychosocial stress. Psychopharmacology (Berl). 2016;233:2459–67. 27087432 10.1007/s00213-016-4299-5 PMC4907902

[20] Heatherton TF, Kozlowski LT, Frecker RC, Fagerström KO. The Fagerström test for nicotine dependence: a revision of the fagerström tolerance questionnaire. Br J Addict. 1991;86:1119–27. 1932883 10.1111/j.1360-0443.1991.tb01879.x

[21] Spielberger CD, Gorsuch RL, Lushene RE. Manual for the state-trait anxiety inventory. Consulting Psychologists Press; 1983.

[22] Paloyelis Y, Doyle OM, Zelaya FO, Maltezos S, Williams SC, Fotopoulou A, et al. A spatiotemporal profile of in vivo cerebral blood flow changes following intranasal oxytocin in humans. Biol Psychiatry. 2016;79:693–705. 25499958 10.1016/j.biopsych.2014.10.005

[23] Dedovic K, Renwick R, Mahani NK, Engert V, Lupien SJ, Pruessner JC. The Montreal Imaging Stress Task: using functional imaging to investigate the effects of perceiving and processing psychosocial stress in the human brain. J Psychiatry Neurosci JPN. 2005;30:319–25. 16151536 PMC1197276

[24] Goodman AM, Wheelock MD, Harnett NG, Mrug S, Granger DA, Knight DC. The hippocampal response to psychosocial stress varies with salivary uric acid level. Neuroscience. 2016;339:396–401. 27725214 10.1016/j.neuroscience.2016.10.002 PMC5118067

[25] Goodman AM, Harnett NG, Wheelock MD, Hurst DR, Orem TR, Gossett EW, et al. Anticipatory prefrontal cortex activity underlies stress-induced changes in Pavlovian fear conditioning. NeuroImage. 2018;174:237–47. 29555429 10.1016/j.neuroimage.2018.03.030 PMC5949265

[26] Cox RW. AFNI: Software for analysis and visualization of functional magnetic resonance neuroimages. Comput Biomed Res. 1996;29:162–73. 8812068 10.1006/cbmr.1996.0014

[27] Cox RW, Hyde JS. Software tools for analysis and visualization of fMRI data. NMR Biomed. 1997;10:171–8. 9430344 10.1002/(SICI)1099-1492(199706/08)10:4/5<171::AID-NBM453>3.0.CO;2-L

[28] Chen G, Saad ZS, Britton JC, Pine DS, Cox RW. Linear mixed-effects modeling approach to FMRI group analysis. NeuroImage. 2013;73:176–90. 23376789 10.1016/j.neuroimage.2013.01.047 PMC3638840

[29] Tzourio-Mazoyer N, Landeau B, Papathanassiou D, Crivello F, Etard O, Delcroix N, et al. Automated anatomical labeling of activations in SPM using a macroscopic anatomical parcellation of the MNI MRI single-subject brain. NeuroImage. 2002;15:273–89. 11771995 10.1006/nimg.2001.0978

[30] McClernon FJ, Addicott MA, Sweitzer MM. Smoking abstinence and neurocognition: implications for cessation and relapse. Curr Top Behav Neurosci. 2015;23:193–227. 25655892 10.1007/978-3-319-13665-3_8

[31] Kogler L, Müller VI, Chang A, Eickhoff SB, Fox PT, Gur RC, et al. Psychosocial versus physiological stress—meta-analyses on deactivations and activations of the neural correlates of stress reactions. NeuroImage. 2015;119:235–51. 26123376 10.1016/j.neuroimage.2015.06.059 PMC4564342

[32] Pobric G, Lambon Ralph MA, Zahn R. Hemispheric specialization within the superior anterior temporal cortex for social and nonsocial concepts. J Cogn Neurosci. 2016;28:351–60. 26544918 10.1162/jocn_a_00902

[33] Schultz J, Friston KJ, O’Doherty J, Wolpert DM, Frith CD. Activation in posterior superior temporal sulcus parallels parameter inducing the percept of animacy. Neuron. 2005;45:625–35. 15721247 10.1016/j.neuron.2004.12.052

[34] Lancaster K, Carter CS, Pournajafi-Nazarloo H, Karaoli T, Lillard TS, Jack A, et al. Plasma oxytocin explains individual differences in neural substrates of social perception. Front Hum Neurosci. 2015; **9**. 10.3389/fnhum.2015.00132. 10.3389/fnhum.2015.00132 PMC4362216 25852519

[35] Liberzon I, Taylor SF, Amdur R, Jung TD, Chamberlain KR, Minoshima S, et al. Brain activation in PTSD in response to trauma-related stimuli. Biol Psychiatry. 1999;45:817–26. 10202568 10.1016/S0006-3223(98)00246-7

[36] Luhar RB, Sawyer KS, Gravitz Z, Ruiz SM, Oscar-Berman M. Brain volumes and neuropsychological performance are related to current smoking and alcoholism history. Neuropsychiatr Dis Treat. 2013;9:1767–84. 24273408 10.2147/NDT.S52298 PMC3836660

[37] Adelhöfer N, Beste C. EEG signal decomposition evidence for a role of perceptual processes during conflict-related behavioral adjustments in middle frontal regions. J Cogn Neurosci. 2020;32:1381–93. 32163322 10.1162/jocn_a_01558

[38] Schurz M, Radua J, Aichhorn M, Richlan F, Perner J. Fractionating theory of mind: a meta-analysis of functional brain imaging studies. Neurosci Biobehav Rev. 2014;42:9–34. 24486722 10.1016/j.neubiorev.2014.01.009

[39] Curtis CE, D’Esposito M. Persistent activity in the prefrontal cortex during working memory. Trends Cogn Sci. 2003;7:415–23. 12963473 10.1016/S1364-6613(03)00197-9

[40] Seeley WW, Menon V, Schatzberg AF, Keller J, Glover GH, Kenna H, et al. Dissociable intrinsic connectivity networks for salience processing and executive control. J Neurosci Off J Soc Neurosci. 2007;27:2349–56. 10.1523/JNEUROSCI.5587-06.2007 PMC2680293 17329432

[41] Shamay-Tsoory SG, Abu-Akel A. The social salience hypothesis of oxytocin. Biol Psychiatry. 2016;79:194–202. 26321019 10.1016/j.biopsych.2015.07.020

[42] Warbrick T, Mobascher A, Brinkmeyer J, Musso F, Stoecker T, Shah NJ, et al. Direction and magnitude of nicotine effects on the fMRI BOLD response are related to nicotine effects on behavioral performance. Psychopharmacology (Berl). 2011;215:333–44. 21243486 10.1007/s00213-010-2145-8 PMC3083509

[43] Neumann ID, Landgraf R. Balance of brain oxytocin and vasopressin: implications for anxiety, depression, and social behaviors. Trends Neurosci. 2012;35:649–59. 22974560 10.1016/j.tins.2012.08.004

[44] Eckstein M, Scheele D, Patin A, Preckel K, Becker B, Walter A, et al. Oxytocin facilitates pavlovian fear learning in males. Neuropsychopharmacology. 2016;41:932–9. 26272050 10.1038/npp.2015.245 PMC4748433

[45] Nasanbuyan N, Yoshida M, Takayanagi Y, Inutsuka A, Nishimori K, Yamanaka A, et al. Oxytocin-oxytocin receptor systems facilitate social defeat posture in male mice. Endocrinology. 2018;159:763–75. 29186377 10.1210/en.2017-00606

[46] Zahn R, Moll J, Krueger F, Huey ED, Garrido G, Grafman J. Social concepts are represented in the superior anterior temporal cortex. Proc Natl Acad Sci USA. 2007;104:6430–5. 17404215 10.1073/pnas.0607061104 PMC1851074

[47] Harrewijn A, Vidal-Ribas P, Clore-Gronenborn K, Jackson SM, Pisano S, Pine DS, et al. Associations between brain activity and endogenous and exogenous cortisol - a systematic review. Psychoneuroendocrinology. 2020;120:104775. 32592873 10.1016/j.psyneuen.2020.104775 PMC7502528

[48] Zhang W, Hashemi MM, Kaldewaij R, Koch SBJ, Beckmann C, Klumpers F, et al. Acute stress alters the ‘default’ brain processing. NeuroImage. 2019;189:870–7. 30703518 10.1016/j.neuroimage.2019.01.063

[49] Baracz SJ, Everett NA, Cornish JL. The impact of early life stress on the central oxytocin system and susceptibility for drug addiction: applicability of oxytocin as a pharmacotherapy. Neurosci Biobehav Rev. 2020;110:114–32. 30172802 10.1016/j.neubiorev.2018.08.014

