# Supplementary material for: The interaction of oxytocin and nicotine addiction on psychosocial stress: an fMRI study
Source: Transl Psychiatry. 2024 Aug 30;14:348. doi: 10.1038/s41398-024-03016-5 (PMC11364850; doi:10.1038/s41398-024-03016-5)
Supplement: Supplementary file 1 — The interaction of oxytocin and nicotine addiction on psychosocial stress: an fMRI study [file 41398_2024_3016_MOESM1_ESM.docx]

**Supporting Information**

**Title:** **The interaction of oxytocin and nicotine addiction on psychosocial stress: an fMRI study**

**Methods and Materials**

***Participants***

All participants were recruited from local universities and nearby communities by postings. A remuneration was paid after the experiments.

***Salivary cortisol analysis***

The changes in salivary cortisol of stress run 1 and stress run 2 were calculated by subtracting baseline from control run 2, which is the closest control run to the stress run 1. Changes between stress runs were measured by calculating the differences between two MIST stress runs.

***Subjective report ratings***

To prevent potential head motion, participants were asked to assign points by gesturing. The changes in subjective stress ratings of stress run 1 and stress run 2 were calculated by subtracting baseline ratings from control run 2, which is the closest control run to the stress run 1. Changes between stress runs were measured by calculating the differences between two MIST stress runs.

***fMRI acquisition***

MRI data were collected on a 3.0 Tesla GE scanner (General Electric Company, Boston, USA). High-resolution brain structural data were acquired using a T1-weighted sequence (repetition time [TR]= 1500 ms, echo time [TE] = 3.2 ms, flip angle = 12°, thickness = 1 mm, field of view [FOV] = 256 mm $\times$ 256 mm, 192 sagittal slices, voxel size = 1 $\times$ 1 $\times$ 1 mm). Functional data were acquired using a T2-weighted echo planar imaging (EPI) sequence with the following parameters: TR = 2000 ms, TE = 30 ms, flip angle = 90°, voxel size = 3 $\times$ 3 $\times$ 3 mm, FOV = 220 mm $\times$ 220 mm, transverse slices = 36, and thickness = 3 mm.

***fMRI data analyses***

The first two volumes of each functional run were removed to allow MRI signal equilibrium. The preprocessing steps included slice timing, head motion correction, spatial normalization, and smoothing (8 mm full-width at half maximum (FWHM) Gaussian kernel). In the head motion correction step, large motion (≥0.3 mm) between time points was censored, and participants with repetition time (TR) censoring over 10% were excluded. The general linear model (GLM) for each participant contained regressors indicating the block of MIST’s control or experimental condition (duration = 2 $\times$120 s) and six motion-correction parameters.

After whole-brain voxelwise repeated-measures ANOVA was done, masks of regions of interest (ROIs) were created using Automated Anatomic Labeling (AAL) atlas. Coordinates of the center of mass of the four brain regions which had Treat $\times$ Group interaction were identified, and the corresponding four regions in AAL were extracted to create the ROI masks. Four brain regions in AAL were labelled Frontal_Sup_medial_R, ParaHippocampal_R, Putamen_R and Temporal _Pole_Sup_R.

After mean beta estimates of the ROIs were extracted, and changes in beta estimates in stress run 1 and 2 were calculated by subtracting the baseline of control run 2. Changes of beta estimates by using beta estimates of control run 1 as baseline or changes of beta estimates by using mean beta estimates of stress run 1 and 2 subtracting the mean beta estimates of control run 1 and 2 were also calculated. Further *post hoc* t-tests were conducted for within-subject and between-group difference detection.

We conducted psychophysiological interaction (PPI) assessment at the whole-brain level. We modeled the preprocessed task functional data of each participant using GLM, which contained regressors indicating MIST’s block time course, the BOLD signal time course from the seed region, the psychophysiological interaction, and six motion-correction parameters. To avoid double dipping, spheres with a radius of 6 mm centered by peak coordinates were generated as masks to extract mean z-score values of functional connectivities, and *post hoc* t-tests were conducted for within-subject and between-group difference detection.

***Associations between neural and behavioral or physiological data***

Pearson correlations were calculated to investigate relationships between beta estimates of the identified brain regions and subjective stress ratings, salivary cortisol or indexes of nicotine addiction. Also, Pearson correlations were calculated to investigate relationships between mean z-scores of functional connectivities and subjective stress ratings, salivary cortisol or indexes of nicotine addiction. To further explore the associations between neural and behavioural data in changes between two stress runs, Pearson correlations were also calculated using data of changes between the two stress runs.

**Results**

***Brain areas revealed significant Treatment*** $\boldsymbol{\times}$***Group interactions***

After calculated changes of beta estimates in stress run 1 and 2 subtracting the baseline of control run 2, a voxel-wise 2 (Treatment: PLC and OXT) $\times$ 2 (Group: HC and SMO) $\times$ 2 (Time: stress run 1 and stress run 2) repeated-measures ANOVA (stress > control) was carried out on the whole brian. The analysis identified significant Treatment $\times$ Group interactions in several brain regions: right anterior superior temporal gyrus, medial frontal gyrus, right lentiform nucleus / right Inferior frontal gyrus and right parahippocampal gyrus (whole-brain significant at a voxel-wise threshold p < 0.001 and a cluster-wise false positive rate (FPR) correction with p < 0.05; Figure S1A).

We then calculated changes of beta estimates by using beta estimates of control run 1 as baseline. A voxel-wise 2 (Treatment: PLC and OXT) $\times$ 2 (Group: HC and SMO) $\times$ 2 (Time: Stress Run 1 and Stress Run 2) repeated-measures ANOVA (stress > control) identified no brain regions with interactions.

We also calculated changes of beta estimates by using mean beta estimates of stress run 1 and 2 subtracting the mean beta estimates of control run 1 and 2, and performed a voxel-wise 2 (Treatment: PLC and OXT) $\times$ 2 (Group: HC and SMO) repeated-measures ANOVA on the whole brain. The analysis identified significant Treatment $\times$ Group interactions in right anterior superior temporal gyrus (cluster of 56 voxels, peak coordinates in the talaraich space: [-32, -14, -25], p<0.001, uncorrected, Figure S1B).


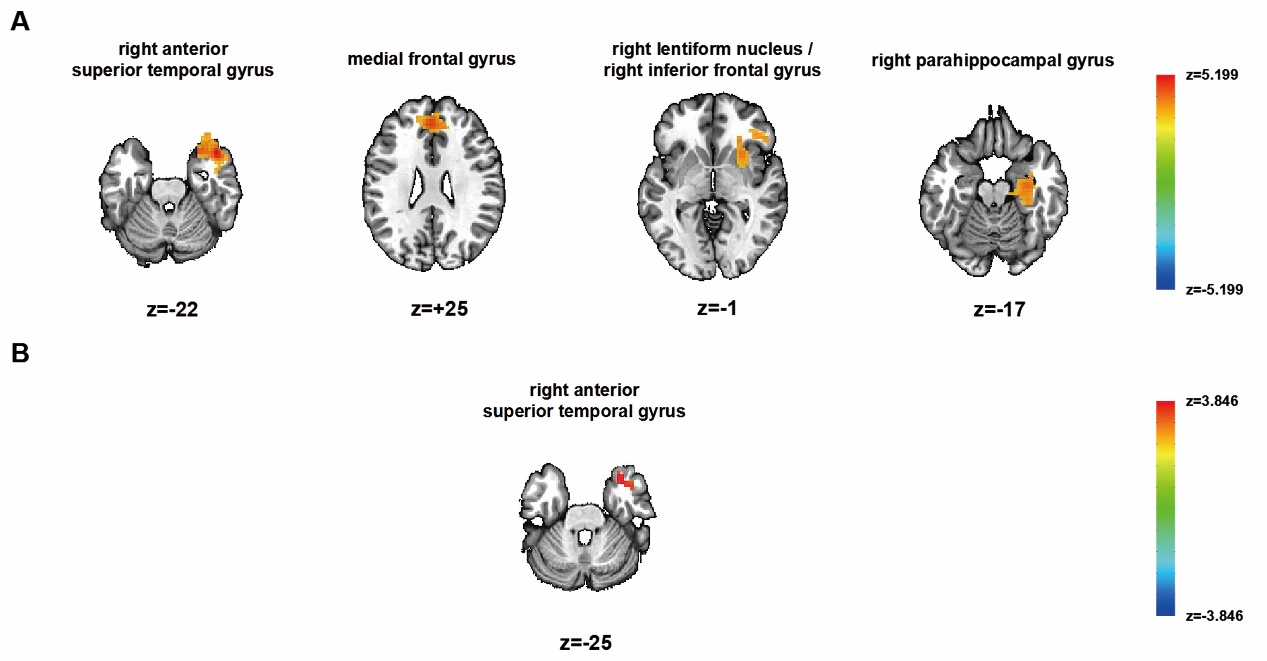


**Figure S1.** Brain areas revealed significant Treatment $\times$Group interaction. This interaction was identified in four brain regions using control run 2 as baseline (A), and was identified in right anterior superior temporal gyrus using mean beta estimates of control run 1 and 2 as baseline (B).

***Neural activity during psychosocial stress in brain areas revealed interaction.***

Neural activity of the four brain regions were extracted by using AAL-defined masks of four brain areas (stress vs. control). Post-hoc examinations of differences were conducted, and results are shown in Figure S2.


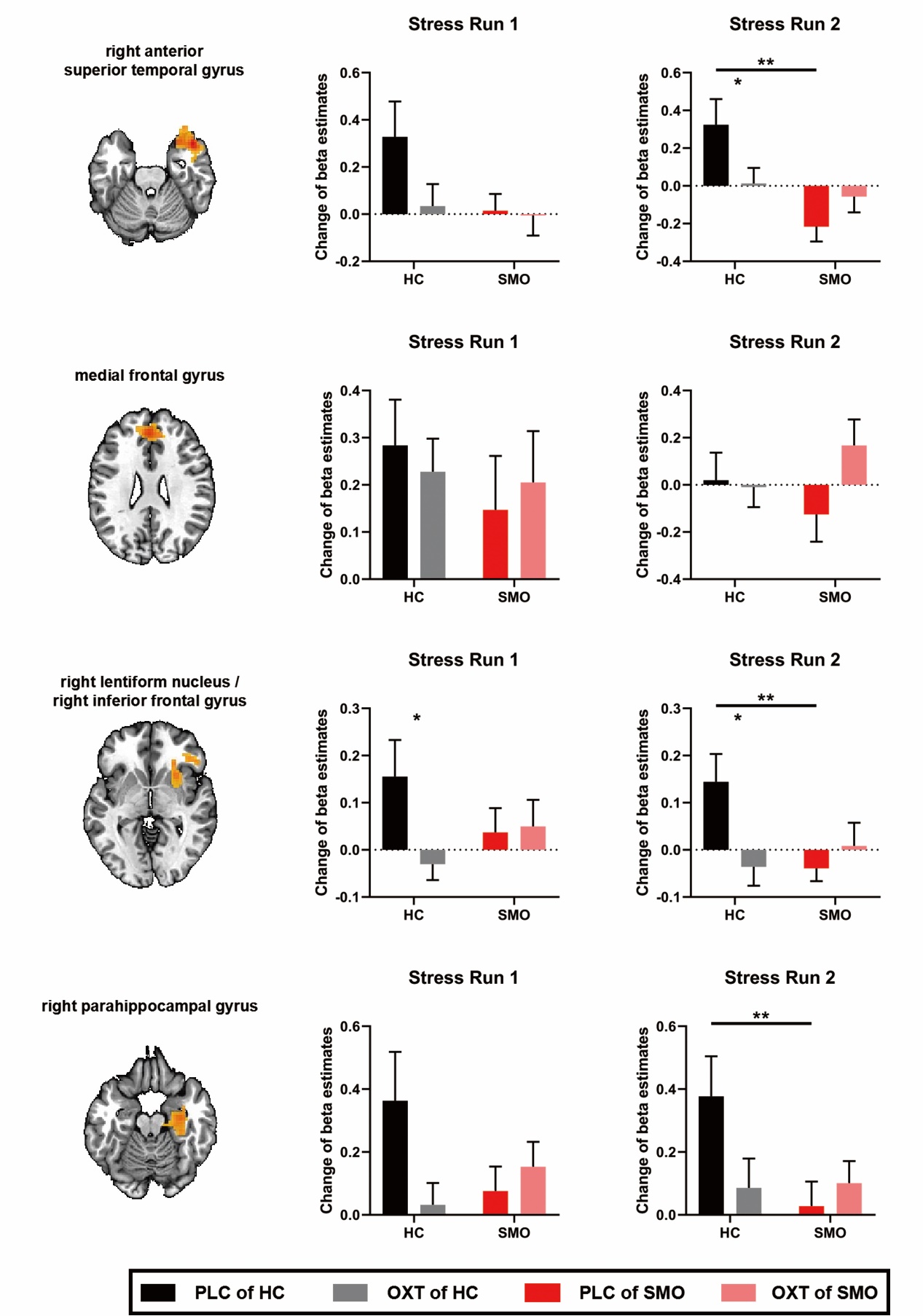


**Figure S2.** Neural activity during psychosocial stress in four brain areas revealed interaction.

***Effects of oxytocin and nicotine on anterior rSTG’s functional connectivities with right precuneus (rPCu).***

In stress run 1, OXT significantly strengthened the connectivity in healthy participants (t_26_=2.670, p=0.013). In Stress Run 2, changes of coupling between anterior right STG and rPCu were significantly differently affected by OXT administration, with OXT significantly strengthened the connectivity in healthy participants (t_26_=-2.976, p=0.006) and significantly weakened the connectivity in smokers (t_25_=-2.223, p=0.036; Figure S3A).

A positive association was observed between changes of salivary cortisol and changes of coupling of anterior rSTG-rPCu after OXT administration when healthy participants and smokers were combined together (r=0.290, p=0.035), which showed a trend of difference with administrated with PLC (z=1.69, p=0.091, two-tailed; Figure S3B).


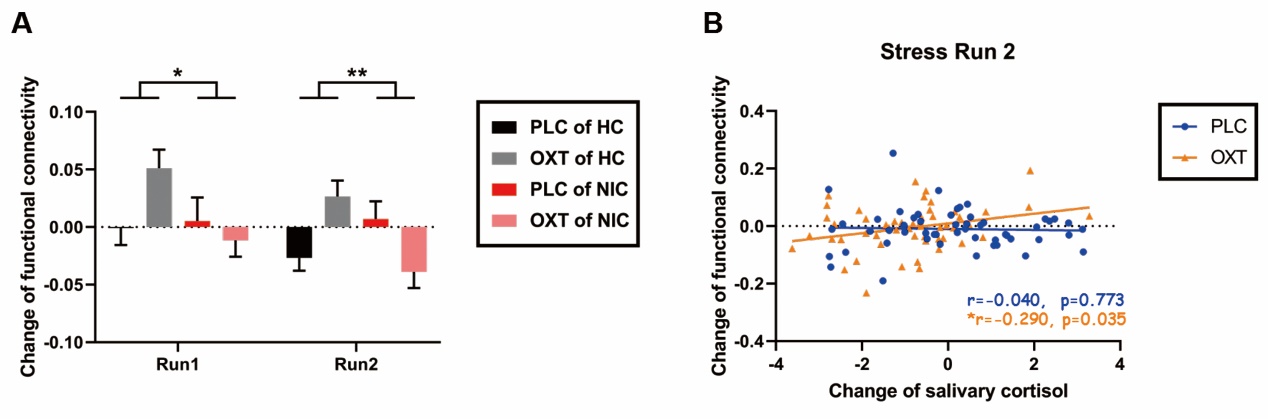


**Figure S3.**  Change of coupling between anterior rSTG and right precuneus (A). A positive association was observed between changes of salivary cortisol and changes of coupling of anterior rSTG-rPCu after OXT administration (B).
